# PhIP-Seq uncovers marked heterogeneity in acute rheumatic fever autoantibodies

**DOI:** 10.1172/jci.insight.196619

**Published:** 2025-11-18

**Authors:** Reuben McGregor, Lauren H. Carlton, Timothy J. O’Donnell, Elliot Merritt, Campbell R. Sheen, Florina Chan Mow, William John Martin, Michael G. Baker, Nigel Wilson, Uri Laserson, Nicole J. Moreland

**Affiliations:** 1 Department of Molecular Medicine, and; 2 Maurice Wilkins Centre for Biodiscovery, The University of Auckland, Auckland, New Zealand.; 3 Department of Genetics and Genomic Sciences, Icahn School of Medicine at Mount Sinai, New York, New York, USA.; 4 Canterbury Health Laboratories, Christchurch, NZ & Biomolecular Interaction Centre, University of Canterbury, Christchurch, New Zealand.; 5 Health New Zealand – Te Whatu Ora, Auckland, New Zealand.; 6 Independent Advisor, Wellington, New Zealand.; 7 Department of Public Health, University of Otago, Wellington, New Zealand.; 8 Starship Children’s Hospital, Health New Zealand – Te Whatu Ora, Auckland, New Zealand.

**Keywords:** Autoimmunity, Cardiology, Antigen

## Abstract

Acute rheumatic fever (ARF) and associated rheumatic heart disease are serious sequelae after infection with group A *Streptococcus* (Strep A). Autoantibodies are thought to contribute to pathogenesis, with deeper exploration of the autoantibody repertoire needed to improve mechanistic understanding and identify new biomarkers. Phage immunoprecipitation sequencing (PhIP-Seq) with the HuScan library (>250,000 overlapping 90-mer peptides spanning the human proteome) was utilized to analyze autoreactivity in sera from children with ARF, uncomplicated Strep A pharyngitis, and matched healthy controls. A global proteome-wide increase in autoantigen reactivity was observed in ARF, as was marked heterogeneity between patients. Public epitopes, common between individuals with ARF were rare, and comprised less than 1% of all enriched peptides. Differential analysis identified both unknown and previously identified ARF autoantigens, including PPP1R12B, a myosin phosphatase complex regulatory subunit expressed in cardiac muscle, and members of the collagen protein family, respectively. Pathway analysis found antigens from the disease-relevant processes encompassing sarcomere and heart morphogenesis were targeted. In sum, PhIP-Seq has substantially expanded the spectrum of autoantigens in ARF, and reveals the rarity of public epitopes in the disease. It provides further support for the role of epitope spreading in pathogenesis and has identified PPP1R12B as an enriched autoantigen.

## Abstract 2

PhIP-Seq profiling in acute rheumatic fever reveals broad, heterogeneous autoantibody responses, identifies novel cardiac autoantigens like PPP1R12B, and supports epitope spreading in pathogenesis.

## Graphical abstract

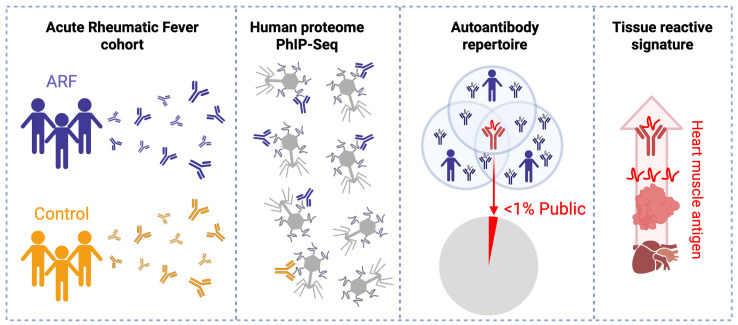

## Introduction

Children with acute rheumatic fever (ARF) present with symptoms consistent with an autoinflammatory disease, including fever, joint pain, subcutaneous nodules, and carditis. These symptoms develop 2–4 weeks after a preceding infection with *Streptococcus pyogenes* (group A *Streptococcus*, Strep A), with the peak incidence age being 5–14 years (1, 2). The pathophysiological link between Strep A throat and skin infections and development of ARF is the subject of ongoing investigation. Molecular mimicry between the coiled-coil M-protein expressed on the surface of Strep A and similarly structured heart proteins such as cardiac myosin have been proposed to trigger disease (3). However, cardiac myosins are intracellular antigens, suggesting that autoantibodies targeting these proteins are unlikely to be sufficient for disease pathology on their own. Other autoantigens, including those that would be more immune accessible, have also been implicated, such as collagens, laminin, and N-acetylglucosamine (GlcNAc) residues on host antigens (1, 3–5), but a definitive, pathological autoantibody trigger remains enigmatic.

The delayed onset of ARF after the precursor Strep A infection suggests a central role for the adaptive immune response in the disease, as antigen-specific T and B cells would require expansion before initiating pathological immune activity. Evidence for a direct role of aberrant adaptive immunity in ARF comes from a combination of pathology and immunophenotyping studies. Immunohistochemistry has identified immune cell infiltrate in rheumatic heart valves with Aschoff bodies, comprising nodules with fibrinoid change and T and B lymphocytes, a hallmark of the disease (6). Antibody and complement deposits have been observed on excised rheumatic valve tissue (7), and stimulation of ARF immune cells with Strep A ex vivo induces release of inflammatory cytokines associated with immune-mediated tissue damage (8). This tissue damage will expose additional antigens and epitopes and enable epitope spreading, a mechanism thought to underpin many autoimmune diseases by driving ongoing immune responses and inflammation (9, 10).

Epitope spreading is increasingly recognized to contribute to heterogeneity in autoantibody responses between individuals with the same autoimmune disease, including rheumatoid arthritis, multiple sclerosis, and systemic lupus erythematosus where patients exhibit unique autoantibody profiles (11–13). Heterogeneity was also observed in our previous study of ARF autoantibodies. Using high-content protein array technology and a small patient cohort, approximately half of the identified IgG reactivity to human antigens was found to be unique to an individual (14). This emphasizes the importance of identifying shared or public epitopes common between individuals. Such epitopes will not only provide insight into pathogenesis but also have biomarker potential. High-content arrays, while comprehensive, do not encompass the full coding potential of the human proteome. Here, we took a more wide-ranging approach by leveraging phage immunoprecipitation and sequencing (PhIP-Seq) technology to explore the autoantibody repertoire in ARF in depth. HuScan PhIP-Seq libraries comprise the entire human proteome represented as peptide tiles, which is combined with a high-throughput sequencing-based output, and has been used to probe the autoantibody repertoire of a range of other autoimmune-mediated diseases (11, 13, 15). By combining PhIP-Seq with a large patient cohort from Aotearoa, New Zealand, a setting with a high prevalence of serious Strep A disease (16), this study substantially expands the spectrum of autoantibody targets in ARF, and reveals the rarity of public epitopes in the disease.

## Results

### Heterogeneous autoantibody repertoire in ARF reveals limited public epitopes.

To gain a global view of the autoantibody response in ARF, PhIP-Seq was performed with the HuScan library, which spans the entire human proteome in overlapping 90-mer peptides (15). The IgG autoantibody profile was examined in a discovery cohort of patients with first-episode ARF (*n* = 52) and matched healthy controls (*n* = 75) (Table 1).

**Figure 1:**
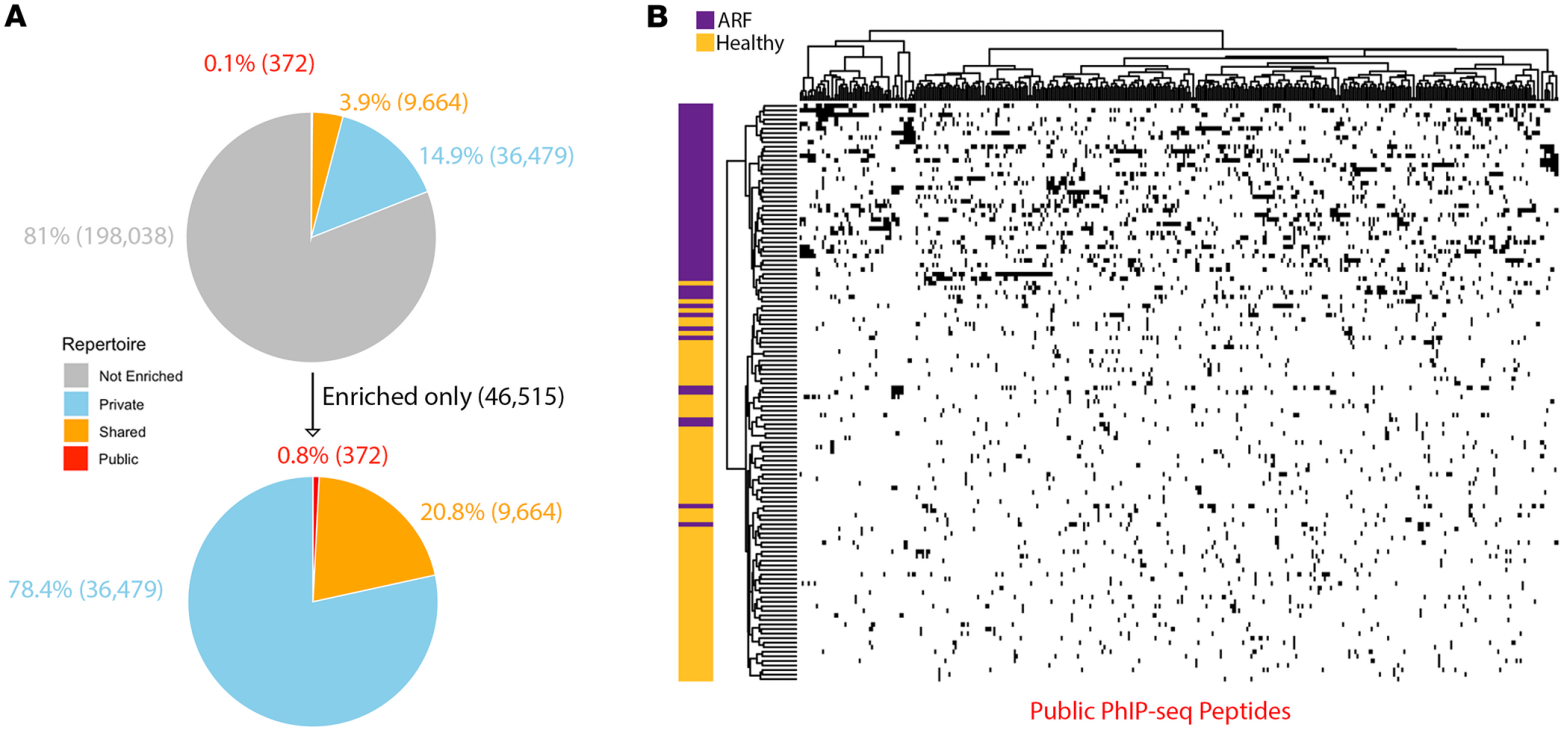
Autoantibody repertoire enrichment. (**A**) All PhIP-Seq peptides were classified based on their enrichment in ARF cases relative to healthy controls using a per-donor *z* score on the VST scale. A peptide was considered enriched for a given case if *z* was 3 or greater versus the control distribution and the 1-sided upper-tail *P* value remained significant after Benjamini-Hochberg adjustment within donor at *q* of 0.20 or less. Peptides were further categorized as Not Enriched (no cases enriched, grey), Private (enriched in a single case, blue), Shared (enriched in <10% of cases, orange), or Public (enriched in ≥10% of cases, red). Pie charts show the distribution of these categories across all peptides (top) and among enriched peptides only (bottom). (**B**) Heatmap showing reactivity to only public peptides (enriched in ≥10% of ARF cases, *n* = 372) across all individuals. Samples are grouped by case status (ARF, *n* = 52; healthy control, *n* = 75) and indicated by a left annotation bar (ARF = purple, control = gold). Columns (donors) and rows (peptides) were hierarchically clustered using a binary distance and average-linkage agglomeration. Each square represents binary reactivity; black = enriched, white = not (enriched based on *z* score ≥ 3 and *q* ≤ 0.1).

Initial analysis aimed to explore similarities and differences in autoantibodies between individuals with ARF. The overall similarity of autoantibody repertoires between individual cases was assessed by identifying peptides with significantly elevated reactivity relative to the healthy control cohort. A peptide was considered enriched in an individual if reactivity was significantly elevated compared with the healthy control cohort (*z* score ≥ 3 relative to the healthy control distribution and within-donor *q* value ≤ 0.1). The vast majority (198,038; 81%) of peptides were not enriched in ARF over healthy controls, with the remaining 46,515 (19%) being enriched in at least one ARF case (Figure 1A). Enriched peptides were further classified as public, shared, or private: “public” peptide if enriched in more than 10% of ARF cases, “shared” if enriched in more than one but fewer than 10% of cases, and “private” if enriched in only one case. Only 372 peptides (0.1% of all peptides and 0.8% of enriched peptides), mapping to 322 proteins, met the criterion for public epitopes (Figure 1A and Supplemental Dataset 1; supplemental material available online with this article; https://doi.org/10.1172/jci.insight.196619DS1), indicating a high degree of heterogeneity between ARF cases with respect to autoantibodies.

The heterogeneity is clearly evident when reactivity against public peptides is viewed as a binary heatmap. Hierarchical clustering of the heatmap segregates most ARF cases from healthy controls, illustrating that the autoantibody profiles are distinct between the groups (Figure 1B). Importantly, clustering does not resolve into distinct subgroups of reactivity within the cases, with each individual displaying a relatively unique barcode of autoreactivities, and no clear pattern of reactivity evident between cases. Some healthy controls also reacted with these public peptides, although the overall level was markedly lower than in ARF (Figure 1B).

### Protein-level analysis reflects the heterogeneous autoantibody signature in ARF.

**Figure 2:**
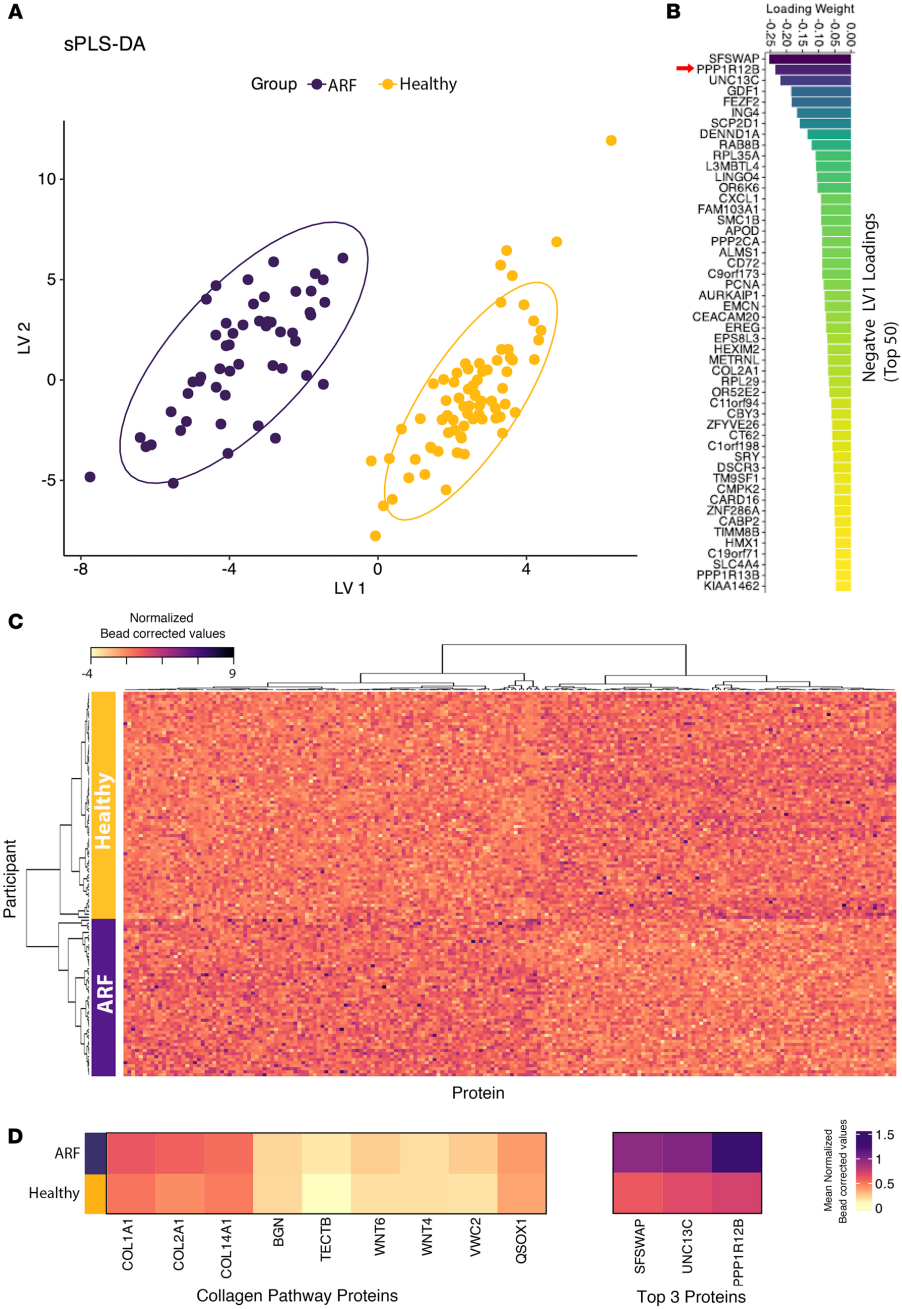
Identification of discriminatory protein-level autoantibodies between ARF and healthy controls. (**A**) Scatter plot of sPLS-DA latent variable 1 (LV1) versus LV2 scores for individual samples. ARF cases (purple) and healthy controls (gold) are separated, with 95% confidence ellipses shown for each group. (**B**) Top 50 negative loading weights on LV1, representing protein-level autoantibodies most strongly associated with ARF cases. Bars are ordered by magnitude and colored according to loading strength. (**C**) Heatmap of protein-level autoantibodies expression for features selected by sPLS-DA across participants. Rows represent individuals colored by case/control status; columns represent selected features. Euclidean distance and complete linkage hierarchical clustering were applied to both rows and columns. (**D**) Heatmap of collagen-associated proteins identified from pathway enrichment analysis of top LV1 features. Rows represent the mean autoantibody reactivity in ARF and healthy control groups, and columns represent autoantibodies targeting collagen-related proteins. Reactivity against the top 3 proteins contributing to LV-10, noncollagen proteins, are included (right) for comparison of autoantibody magnitude. Color intensity reflects relative autoantibody reactivity.

ARF is clinically heterogeneous, with a range of symptoms and disease severity (9). To further explore the autoantibody heterogeneity observed above and in prior work (14), the autoantibody repertoire was examined at the protein level. While the initial peptide-level analysis revealed the scale of private autoreactivity, aggregating these signals to the protein level allows exploration of how these diverse targets could be combined to create a disease-specific signature. The goal was to explore the utility of multivariate modeling to generate a broad, protein-level classifier that distinguishes ARF cases from healthy controls and reflects clinical heterogeneity. To account for varying antigen size, protein-level reactivity was determined by averaging the top 4 peptide signal intensities for each protein. The resulting protein-level matrix (18,769 proteins) was analyzed with sparse partial least squares discriminant analysis (sPLS-DA) to identify a minimal set of autoantibodies discriminating ARF cases from healthy controls in the discovery cohort. A set of 300 proteins (200 on latent variable 1 [LV1] and 100 on LV2, Supplemental Table 1) yielded clear separation in the sPLS-DA projection. This requirement for hundreds of proteins is consistent with a distributed, partially shared signal within a background of person-to-person variability (Figure 2, A and B).

A heatmap of all the 300 proteins selected in the sPLS-DA shows cohort-consistent grouping of ARF and controls by unsupervised hierarchical clustering, with substantial within-group heterogeneity (Figure 2C). Pathway enrichment analysis (17), utilizing the LV1 proteins for which autoantibodies were elevated in ARF compared with controls (*n* = 171), revealed a top-ranked cluster of significantly enriched, closely related terms associated with collagen biology (*P* < 0.0001, Supplemental Table 1). When the autoantibody reactivity of all proteins in this pathway that were identified in the sPLS-DA was compared, clear elevation in ARF was observed compared with controls (Figure 2D and Supplemental Figure 1), with the majority (6 of 9) reaching statistical significance. However, the magnitude of reactivity for individual proteins in this collagen biology pathway was lower than that observed for the top 3 proteins detected overall (Figure 2D and Supplemental Figure 1).

These results indicate that large, high-dimensional protein sets (hundreds of proteins) are required to capture cohort-level differences between cases and controls. This likely reflects the heterogeneous nature of autoantibody responses in ARF, which is compounded by the inherent heterogeneity in autoantibody repertoires known to be present in healthy individuals (18, 19). The identification of low-level but broad autoreactivity to collagen pathways points to a diffuse autoantibody signature against this abundant protein family that may contribute to disease pathogenesis.

### Elevated autoantibodies in ARF target heart muscle antigens and sarcomere components.

**Figure 3:**
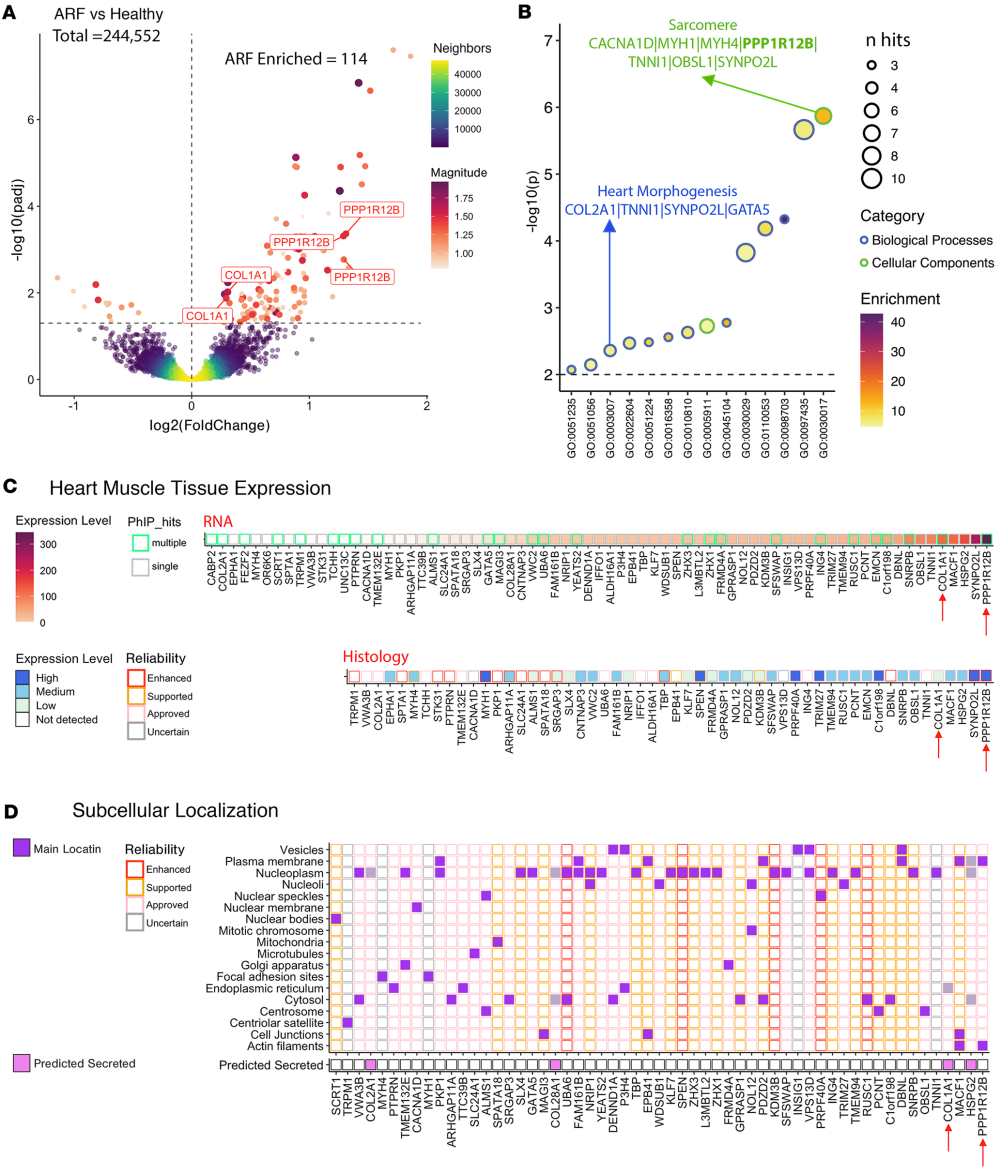
Autoantibody pathway, tissue expression, and localization analysis. (**A**) Volcano plot of differential autoantibody reactivity (PhIP-Seq peptides) between ARF cases and healthy controls. Significant peptides (using DESeq2, adjusted *P* < 0.05) colored by average binding magnitude, nonsignificant by sample density. Number of peptides significantly elevated in ARF cases compares with healthy controls is indicated; 118 peptides mapping to 81 proteins. Dashed lines indicate thresholds for significance and no change; enriched targets PPP1R12B and COL1A1 labeled. Total magnitude indicated by red scale; ARF significantly elevated magnitude compared with healthy controls by 1-sided Wilcoxon’s test. *P* = 0.018, *r* = 0.35. (**B**) GO pathway enrichment for ARF-associated autoantigens (*P* < 0.01, 81 proteins represented by 118 peptides in **A**). Dot size reflects protein count per pathway; fill color indicates enrichment strength. Outlines denote GO categories: Cellular Component (blue), Biological Process (green). Highlighted pathways include Sarcomere and Heart Morphogenesis. Full pathway analysis results with enrichment metrics for all GO terms are provided in Supplemental Dataset 1. (**C**) Heart muscle expression of ARF-enriched targets (Human Protein Atlas). Upper panel: RNA expression ordered from low to high; tile borders indicate multiple (green) or single (gray) peptide hits. Lower panel: Protein expression by immunohistochemistry; tile borders indicate staining reliability (enhanced, red; supported, orange; approved, pink; uncertain, gray). Red arrows highlight PPP1R12B and COL1A1. (**D**) Subcellular localization and secretion prediction (Human Protein Atlas). Upper panel: Localization across cellular compartments (purple = presence); borders show reliability. Lower panel: Secreted proteins prediction (violet = predicted secreted). Main locations shaded if proteins predicted secreted as main location. PPP1R12B and COL1A1 indicated by red arrows.

The initial peptide analysis and protein-level multivariate modeling confirmed marked heterogeneity in the ARF autoantibody response, with a limited number of shared or public epitopes between individuals. To best identify peptides with consistently elevated enrichment across the ARF group, a statistically rigorous differential analysis was performed (using DESeq2). In total, 114 peptides mapping to 81 proteins were identified as being significantly enriched (adjusted *P* < 0.05, fold change > 1) in ARF sera compared with healthy controls, with 50 of these peptides also classified as public in the prior analysis (Figure 3A and Supplemental Dataset 1). In contrast, there were only 13 peptides (mapping to 10 proteins) that were enriched in healthy controls, indicating a general increase in the breadth of autoantibodies in ARF (Supplemental Dataset 1). An increased magnitude in autoantibodies was also observed in ARF, with higher reactivity against target peptides compared with healthy controls (1-sided Wilcoxon’s test, *P* = 0.018; *r* = 0.35; total magnitude indicated by the red scale in Figure 3A). The validity of the analysis approach was confirmed by the identification of both myosin heavy chain (MYH1 and MYH4) and collagen (type 1, α1 [COL1A1], COL2A1, and COL28A1) subunits as being enriched in ARF (Supplemental Dataset 1). Autoantibodies against members of both of these protein families have been previously associated with ARF pathogenesis (14, 20, 21).

Pathway analysis of the 81 elevated autoantigens revealed 14 significantly overrepresented (*P* < 0.01) Gene Ontology (GO) pathways (Figure 3B and Supplemental Dataset 1). In keeping with the protein-level analysis, collagen-related pathways were also captured in this peptide-level analysis. This is indicated within the “supramolecular fiber organization” cluster (Figure 3B and Supplemental Dataset 1), which aggregates terms such as “fibrillar collagen trimer” and “banded collagen fibril.” The “sarcomere” cellular component was the most significant (*P* < 0.001, enrichment score = 12.7) and was represented by 7 proteins, including 2 myosin heavy chain isoforms (MYH1 and MYH4) and protein phosphatase 1 regulatory subunit 12B (PPP1R12B) (Figure 3B and Supplemental Dataset 1). The “heart morphogenesis” biological process was also significantly overrepresented in the pathway analysis. Given the suggested involvement of autoantibodies targeting heart muscle, tissue expression of the hits was explored using data from the Human Protein Atlas (HPA) (22). Of all 81 ARF-enriched autoantigens, PPP1R12B showed the highest RNA expression in heart muscle tissue and had a high expression level by histology with enhanced reliability (highest certainty level) (Figure 3C). Of note, PPP1R12B was also highly ranked in the previous protein-level analysis (second loading on LV1, Figure 2B). The collagen subunit COL1A1 showed the fifth highest RNA expression in heart muscle tissue, but lower approved histology staining (Figure 3C). Next, the subcellular localization data of the target peptides was extracted from the HPA. Four of the 57 proteins were predicted to be secreted, while most were predicted as localizing to intracellular compartments such as the cytosol and nucleoplasm (Figure 3D). This suggests these proteins may be secondary targets in ARF, with their exposure resulting from immune-mediated tissue damage and epitope spreading. In contrast, PPP1R12B is annotated in the HPA with “approved” evidence for localization to both the plasma membrane and actin filaments (23), while COL1A1 is a known extracellular protein and key component of the extracellular matrix (24) (Figure 3D). These localization data suggest that PPP1R12B and COL1A1 might serve as primary or early autoantigens in ARF pathogenesis.

### PhIP-Seq hit exploration and validation.

**Figure 4:**
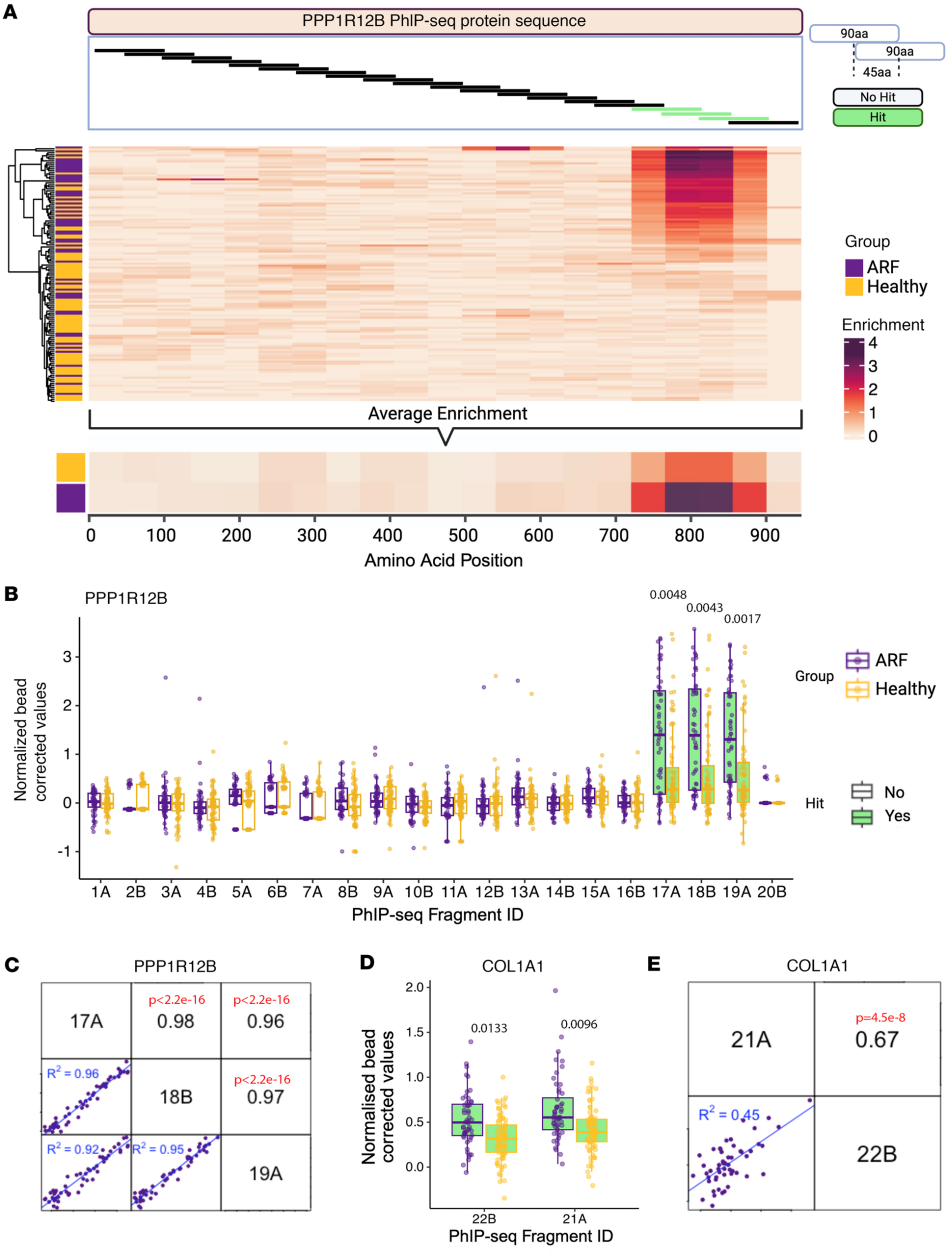
Epitope mapping and characterization of PhIP-Seq hits. (**A**) Top: Schematic of PhIP-Seq peptide tiling for PPP1R12B protein; significant peptides (green) cluster at C-terminus. Middle: Heatmap of normalized, bead-corrected enrichment across PPP1R12B for ARF cases (purple) and controls (gold), clustered by donor profiles. Bottom: Mean enrichment for ARF and controls. (**B**) Individual peptide enrichment for PPP1R12B peptides in ARF (purple) versus controls (gold); significantly enriched peptides (17A, 18B, 19A) highlighted green with *P* values from 2 analysis shown in black text above. (**C**) Correlation of enriched PPP1R12B peptides in ARF. Upper panels: Pearson’s correlation coefficient (*r*) with *P* values from the regression show in red text. Lower panels: Scatterplots with regression lines and *R*^2^ values. (**D**) Enrichment of COL1A1 peptides in ARF (purple) and controls (gold). (**E**) Correlation analysis between COL1A1 peptides in ARF. Upper panel: Pearson’s correlation coefficient (*r*) with *P* values from the regression shown in red text. Lower panels: scatterplots with regression lines and *R*^2^ values. Box-and-whisker plots show median (line in box), interquartile range (IQR, box bounds), and 1.5 × IQR (whiskers).

Building on the identification of PPP1R12B as an autoantibody target in ARF and COL1A1 as a previously implicated antigen (14), these antigens were subject to further characterization and validation. PPP1R12B is a myosin phosphatase regulator preferentially expressed in the heart, skeletal muscle, and brain, and has been implicated in cardiovascular pathophysiology (25). In the HuScan library, PPP1R12B is represented by 21 tiled 90-mer peptides, with 45–amino acid overlaps. The 3 peptides significantly enriched in ARF mapped to a continuous 180–amino acid sequence in the C-terminal region (Figure 4, A and B). Normalized read data visualized as a heatmap across the full protein sequence revealed a distinct immunodominant region. Unsupervised hierarchical clustering partially segregated ARF cases from controls, with cases exhibiting higher enrichment in this antigenic region (Figure 4A). Normalized read data showed significantly elevated reactivity to these 3 peptides (17A, 18B, and 19A) in ARF, with all other peptides showing similar reactivity to healthy controls (Figure 4B). Enrichment for 3 overlapping peptides was significantly and strongly correlated in ARF (Pearson’s *r* > 0.9, *P* < 0.001; Figure 4C). Similar trends, albeit less pronounced, were seen in the 2 overlapping COL1A1 peptides. Reactivity to these peptides was higher in ARF cases than controls, with reactivity between the 2 peptides correlating (Figure 4, D and E, and Supplemental Figure 2A). It is interesting to note that PPP1R12A, a protein from the same family and with 87% sequence homology to PPP1R12B (26), showed no significant enriched autoantibody binding in ARF (Supplemental Figure 2B).

**Figure 5:**
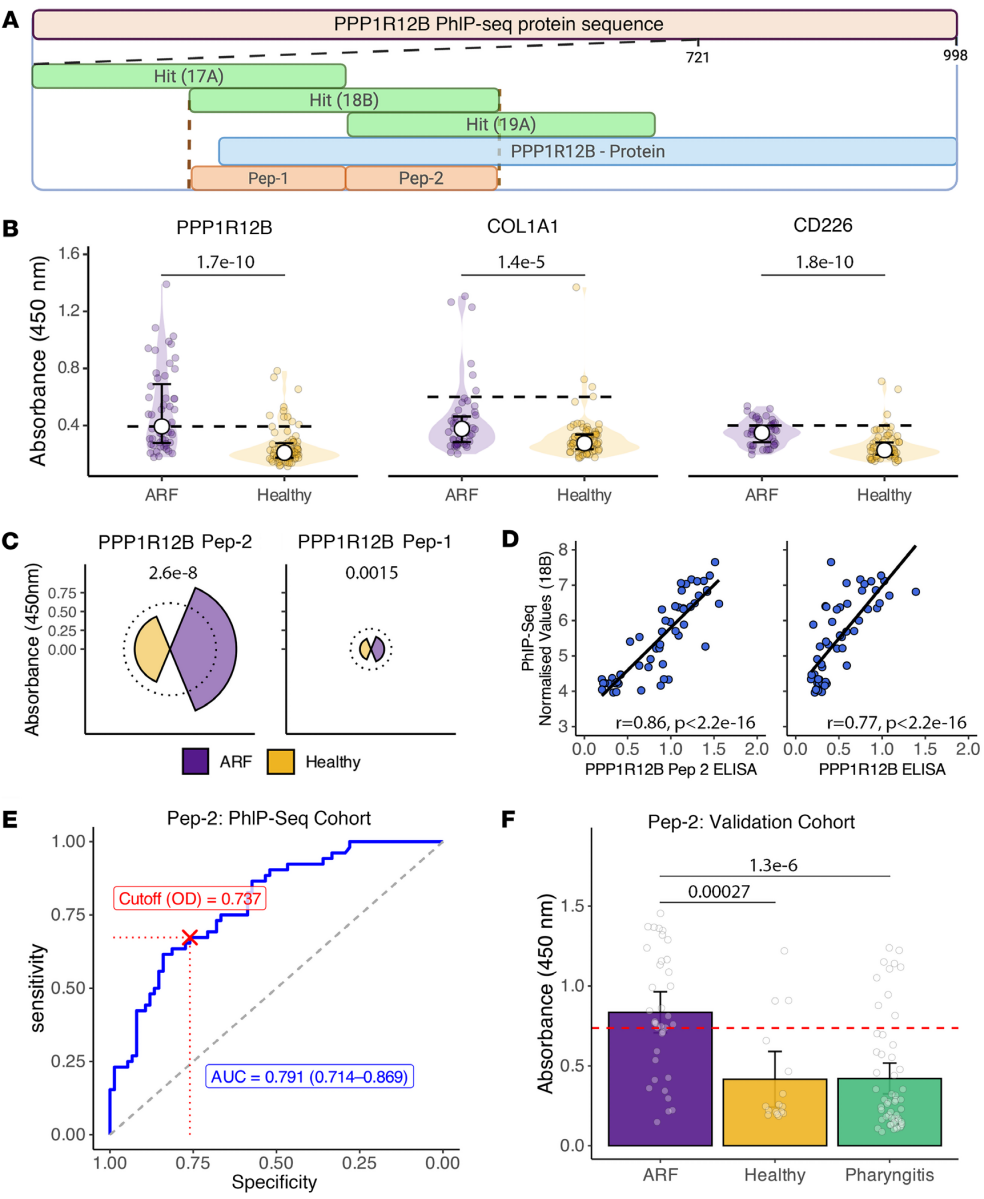
Orthogonal validation using ELISA. (**A**) Schematic representation of the PPP1R12B PhIP-Seq peptide tiling and design of synthetic peptides used for validation. The 3 overlapping PhIP-Seq peptides significantly enriched in ARF (17A, 18B, 19A) localize to a contiguous region of the protein. Two nonoverlapping 45-mer peptides (Pep-1 and Pep-2) were synthesized spanning this region, with Pep-2 fully contained within the recombinant protein used for whole-protein ELISA. Created with Biorender. (**B**) Whole-protein ELISA responses to PPP1R12B, COL1A1, and CD226 in the discovery cohort (ARF vs. healthy). Violin plots with points; white circle symbols show the median, with IQR as error bars. Horizontal dashed lined indicate the reference threshold of 2 times the median of healthy controls. (**C**) Median absorbance in PPP1R12B peptide ELISAs in the discovery cohort; dashed lines indicate the reference threshold of 2 times the median of healthy controls. Wilcoxon’s *P* values (ARF vs. healthy) are shown per peptide. (**D**) Spearman’s correlation of PhIP-Seq normalized enrichment values for peptide 18B with ELISA absorbance values for PPP1R12B whole protein and Pep-2 with fitted linear regression lines (black) and Spearman’s correlation coefficient with significance shown in black text. (**E**) Discovery ROC for Pep-2 on the absorbance scale (ARF vs. healthy). The prespecified operating cutoff is marked in red with dotted guides. AUC and 95% CI are shown on the plot. (**F**) Independent validation cohort (ARF, healthy, and Strep A–positive pharyngitis controls): distribution of Pep-2 absorbance with the prespecified discovery cutoff (red dashed line). Wilcoxon’s *P* values are shown throughout in black text.

To orthogonally validate the 2 hits, a series of ELISA-based approaches were used. First, whole-protein ELISAs incorporating recombinant PPP1R12B and COL1A1 were performed using sera from the same discovery cohort used in PhIP-Seq. This stage also included an additional antigen, CD226, identified using earlier analyses. For PPP1R12B, the recombinant protein represents a C-terminally spliced protein of the larger protein represented in HuScan. This splice variant includes the vast majority of the sequence for 2 of the 3 peptide hits (19A and 18B) (Figure 5A), while the COL1A1 and CD226 antigens comprise the entire protein sequence represented in HuScan. In whole-antigen ELISA, there was significantly elevated reactivity of ARF serum to all 3 proteins. However, only PPP1R12B showed a substantive mean increase in binding in ARF above a defined cutoff (2 × median of the healthy controls) (Figure 5B), warranting further investigation with respect to biomarker potential.

To further validate the PPP1R12B autoreactivity and define the antigenic region, 2 sequential, nonoverlapping 45-mer peptides were designed and synthesized (PPP1R12B Pep-1 and Pep-2), covering the central 90 amino acids of the 3 overlapping peptides identified (Figure 5A). Peptide ELISAs showed significant elevation in ARF above the cutoff (2 × median of the healthy controls) for PPP1R12B Pep-2 only, suggesting this 45-mer is the major target for autoantibodies (Figure 5C). This is corroborated by the whole-protein ELISA with the recombinant antigen containing 80% (36 out of 45 amino acids) of Pep-1 compared with the entirety of Pep-2. To confirm orthogonal validation of PPP1R12B, ELISA data were correlated with normalized read counts of peptide 18B in PhIP-Seq. Both the whole-protein ELISA and PPP1R12B Pep-2 reactivity were well correlated with 18B read counts (Figure 5D), with the stronger correlation for the peptide likely reflecting the peptide-based nature of PhIP-Seq.

Finally, to evaluate Pep-2 as a diagnostic tool, a receiver operating characteristic (ROC) curve was generated based on the ELISA results in the discovery cohort (PhIP-Seq, ARF vs. healthy) to identify a prespecified cutoff (Figure 5E). This cutoff was then applied to an independent validation cohort that included ARF and both healthy children and those with uncomplicated Strep A–positive pharyngitis to provide a more clinically realistic control group (Supplemental Table 1). In this validation cohort, Pep-2 distinguished ARF from combined controls with a sensitivity of 0.667 (95% CI 0.500–0.806), specificity of 0.814 (95% CI 0.714–0.900), and accuracy of 0.764 (95% CI 0.679–0.840); the AUC was 0.804 (95% CI 0.718–0.889) (Figure 5F).

## Discussion

This investigation of the autoantibody repertoire in ARF using PhIP-Seq has revealed marked heterogeneity, characterized by highly individualized autoantibody profiles and limited public epitopes, consistent with prior studies employing protein arrays (14). The protein-level analysis showed that while discrimination between ARF cases and matched healthy controls is possible in this cohort with autoantibodies alone, it requires a large number of variables. This highlights the complex nature of the disease and creates a central challenge — to determine whether specific, pathogenic signals can be distinguished from the broad, individualized inflammatory backdrop of the disease. A more clinically applicable diagnostic strategy will likely require the integration of key autoantibodies, such as those targeting PPP1R12B, with markers of inflammation and prior streptococcal infection. ARF is currently diagnosed using the Jones criteria (or modifications thereof), which include C-reactive protein as a marker of active inflammation and streptococcal serology (anti–streptolysin O and anti–DNase B) as evidence of prior infection (27, 28). The addition of autoantibody markers to C-reactive protein and streptococcal serology may improve diagnosis, given that autoantibodies represent a disease feature distinct from inflammation and the preceding Strep A infection. Consistent with this view, PPP1R12B distinguished ARF from healthy and Strep A pharyngitis controls with encouraging performance, but it may be better suited as a component of a multimarker approach rather than a stand-alone diagnostic.

The pronounced diversity in autoantibodies detected using PhIP-Seq further supports the role of epitope spreading as a driver of immune-mediated tissue damage in ARF. Indeed, epitope spreading, whereby antigens beyond those that initially trigger the autoimmune response are targeted as tolerance breaks down (13, 29), may also contribute to the heterogeneity and diverse clinical manifestations of the disease. The enrichment of autoantibodies targeting collagen pathways in ARF observed in the protein-level analysis exemplifies these mechanisms. Diffuse but elevated responses were observed to multiple collagens, including COL1A1, COL2A1, and COL14A1. Given the known involvement of connective tissue in ARF pathogenesis, affecting not only the heart valves but also joints and skin (1, 20), broad low-level reactivity against the abundant collagen protein family may contribute to the systemic inflammatory nature of the disease, and is consistent with prior studies of collagen reactivity in ARF (21, 30).

Despite this widespread heterogeneity, a compelling finding from this study was the identification of PPP1R12B as an ARF autoantigen. Disease relevance is supported by predominant expression in cardiac and skeletal muscle where it regulates myosin phosphatase activity and muscle cell contractility (31–33). In contrast, PPP1R12A, a closely related isoform not detected in this study, has a broader tissue distribution and is most abundant in smooth muscle cells (25, 31). The preferential targeting of PPP1R12B, with its prominent role in cardiac muscle function, is of relevance given the cardiac involvement in ARF. This is further supported by a predicted localization to the plasma membrane that may make the antigen more accessible to circulating autoantibodies compared with purely intracellular antigens via homeostatic processes such as exocytosis (34), a possibility that could be explored via antigen localization studies in relevant tissues. Indeed, the identification of both the sarcomere and heart morphogenesis pathways in the peptide analysis point to a general role for autoantibodies that target cardiac antigens as contributing to immune-mediated damage in ARF. Previous work has also highlighted the relevance of specific myosin peptides as targets in ARF (35).

While this PhIP-Seq analysis has provided insights into the autoantibody landscape of ARF, there are several limitations inherent with the technology (11, 36, 37). The focus on linear peptide epitopes means autoantibodies targeting conformational or posttranslationally modified epitopes will not be captured. Additionally, autoantibodies against nonprotein antigens such as carbohydrates, including the previously reported reactivity with GlcNAc residues (38, 39), cannot be assessed with this technology. Future studies employing complementary techniques capable of interrogating both conformational and nonprotein antigens will be crucial for a more comprehensive understanding of the autoantibody repertoire in ARF.

In conclusion, this study highlights the highly heterogeneous and individualized nature of the autoantibody response in ARF. The identification of PPP1R12B as a frequently targeted autoantigen with specific relevance to cardiac tissue, coupled with the diffuse reactivity toward collagens, provides valuable insights into the pathogenesis of this disease. The potential of PPP1R12B Pep-2 as a diagnostic biomarker following orthogonal validation warrants further investigation.

## Methods

### Sex as a biological variable

This study examined both male and female participants.

### Patients and cohorts

Sera were obtained from participants in 2 related studies conducted in Aotearoa, New Zealand. ARF cases included children who presented to hospital with their first episode of ARF and were diagnosed according to the New Zealand modification of the Jones criteria (Table 1) (40). These participants, together with healthy controls closely matched to the ARF cases by age, sex, ethnicity, and socioeconomic status, were recruited as part of the Rheumatic Fever Risk Factor study (RF RISK, 2014–2016) (41). Children with uncomplicated Strep A pharyngitis were recruited as part of a study of Strep A skin and throat infections in school-age children in the Auckland region (2018–2019) (42). Two nonoverlapping cohorts were defined from these studies. First, a discovery cohort was used for PhIP-Seq that comprised ARF cases and matched healthy controls (Table 1). Secondly, an independent validation cohort was used to evaluate diagnostic performance of the selected autoantigenic peptide PPP1R12B. The validation cohort comprised a distinct set of ARF cases, matched healthy controls, and those with Strep A pharyngitis (Supplemental Table 1).

### PhIP-Seq

#### Experimental design and IP protocol.

The screening performed with the HuScan PhIP-Seq library followed previously published protocols (37). The HuScan phage library utilized consisted of 259,345 overlapping 90-aa linear peptides (15). The total IgG concentration of each serum sample was quantified to normalize input into the phage IP reactions that were carried out in duplicate. After an overnight incubation at 4°C, protein A and protein G magnetic Dynabeads (Invitrogen) were added to each reaction for 4 hours with rotating at 4°C. This was followed by 3 washes and addition to PCR master mix containing Q5 polymerase (New England BioLabs). After 15 cycles, the PCR product was added to a second PCR of 20 cycles to add Illumina P5/P7 adapters, and sample barcodes and products were sequenced using published primers (15). PhIP-Seq was performed using *n* = 52 ARF sera and *n* = 75 healthy control sera from the RF RISK Study.

#### PhIP-Seq analysis.

PhIP-Seq reads were aligned using Bowtie2 (43) to the HuScan library sequences to generate a matrix of read counts per peptide in each patient sample. These read-count matrices and patient metadata were read into the R (44) environment (version 4.4.1) using R-studio (45) (version 2024.09.0+375). To exclude peptides without detectable signal above negative controls, a per-peptide fold change relative to bead-only wells was calculated (FC_beads = mean normalized counts in samples ÷ mean in bead-only, calculated within plate). Peptides with FC_beads greater than 1 in fewer than 10 samples were removed from downstream analysis.

For the initial landscape analysis to define enriched peptides on a per-donor basis, a 2-tiered statistical approach was used. First, for each peptide, the mean and standard deviation (SD) of the variance-stabilized, bead-corrected read counts were estimated from the healthy controls (*n* = 75). Per donor, a *z* score was computed for every peptide relative to this control distribution; peptides with zero/undefined control variance used a floor SD set to the fifth percentile of the nonzero control SDs. Second, 1-sided (right-tail) *P* values were derived from the *z* scores and adjusted within donor for multiple testing using the Benjamini-Hochberg (BH) method. A peptide was defined as enriched in an individual if the *z* score was 3 or greater with a BH-adjusted *P* (*q* value)of 0.10 or less.

In the primary analysis, following the removal of nonspecific hits, data were processed using the DESeq2 (46) (version 1.44.0) package, similar to a previously published approach (15). The DESeq2 design formula included both batch and group to adjust for potential confounding batch effects while analyzing differences between cases and controls, generating a table of differentially enriched peptides with associated fold change and adjusted *P* values For downstream analyses and visualizations, variance stabilizing transformation (VST) was used (within DESeq2), followed by batch removal using “removeBatchEffect” in limma (version 3.60.4) (47) and subtracting the mean bead-only values per peptide, producing a normalized bead–corrected value. Among DESeq2-significant peptides, group-specific magnitude was defined as the mean batch-corrected VST count within the ARF group for ARF-enriched peptides and within the control group for control-enriched peptides, compared using a 1-sided Wilcoxon’s rank-sum test with rank-biserial *r* reported. HPA data were accessed and processed using HPAanalyze (48). An earlier analysis approach was used to identify 1 antigen (11). In this approach, the PhIP-Seq read counts per million (CPM) matrix was analyzed using the call-hits command in the phip-stat package (https://github.com/lasersonlab/phip-stat). Briefly, a background distribution of CPM values was generated for each phage clone based on the beads-only (no serum) controls. The CPM value for each serum sample was compared to this distribution individually, and if the CPM value exceeded a percentile threshold, then the clone was identified as a hit. The command used was “phip call-hits -i cpm.tsv -o results.tsv --fdr 0.05.”

#### Protein-level analysis.

To account for differences in antigen size, a top-4 summary approach was applied. For each sample and protein, preprocessed peptide values (variance stabilization and bead correction) were ranked and the mean of the top 4 values calculated as the protein score; proteins represented by fewer than 4 peptides were summarized by the mean of all available peptides. Proteins annotated with a locus “LOC” identifier were excluded prior to sPLS-DA, as these represent uncharacterized or computationally predicted proteins lacking functional annotation. To determine whether the peptide representation method inflated group differences, the absolute case-control difference in top-4 protein scores was correlated with the number of peptides per protein (Spearman). The resulting association was small and negative (ρ = –0.13, *P* = 1.53 × 10^–76^), suggesting no inflation. This yielded protein-level autoantibody scores for 18,769 proteins. sPLS-DA was implemented with the number of components and the number of autoantibodies to retain on each component tuned using cross-validation (10-fold repeated 10 times) to minimize the balanced error rate (BER) using the mixomics package (49). This method combines feature selection and classification by applying L1 (lasso) penalization to the loading vectors of the predictor matrix (*X*), effectively identifying a subset of discriminative variables while constructing latent components that capture the covariance between *X* and the response variable (*Y*). The centroid distance was used as the distance metric for classification during the cross-validation process. An *X* tuning grid was specified to explore different levels of sparsity, including retention of 5–10 variables (increments of 1), 20–100 variables (increments of 10), 200–10,000 variables (increments of 100), and larger models retaining 15,000 or all 18,769 features. This ensured a comprehensive search across sparse and dense models. Based on the tuning results, the optimal model parameters were determined to be 2 latent variables (LVs) with a specified number of autoantibodies retained on each component. A final sPLS-DA model was then constructed using these parameters (2 LVs with 200 variables retained on LV1 and 100 on LV2) to classify samples based on protein-level autoantibodies (49).

#### GO pathway analysis.

Pathway analysis was carried out using Metascape, using custom analysis for enrichment in GO biological processes and cellular components (17). The HuScan PhIP-Seq library spans the entire human proteome; thus, the default of all annotated human genes is appropriate and unbiased relative to the assayed search space. The plotted “Enrichment” is the enrichment factor (EF), which quantifies the fold increase in pathway members compared with expected that by chance: EF = (*n*/*M*)/(*k*/*N*), where *N* is the size of the background set, *k* the number of genes annotated to the GO term/pathway, *M* the size of the input list, and *n* the overlap between the input list and the term. Metascape redundancy reduction was applied with default settings; plotted labels correspond to the representative term of each enriched cluster; full member terms are provided in Supplemental Data 1.

#### Antigenic region mapping.

To visualize autoantibody enrichment across the full PPP1R12B protein sequence, a positional mapping approach was developed. PhIP-Seq normalized enrichment values (bead-corrected and variance-stabilized, with negative enrichment values set to zero before averaging) were assigned to their corresponding amino acid positions based on peptide-protein alignments. Overlapping peptides were handled by summing the enrichment scores across the matching sequence regions and dividing by the number of overlapping peptides to generate a per-position average enrichment score. For the per-sample heatmap, individual donor enrichment scores were mapped to amino acid positions following the same approach, and rows (donors) were hierarchically clustered using Euclidean distance and complete linkage. For the group-level (ARF and healthy) enrichment plots, per-position enrichment values were calculated as the arithmetic mean across all donors within each group. Specifically, for each amino acid position, enrichment values were computed as the mean of bead-corrected, normalized PhIP-Seq enrichment scores. Negative values were set to zero before mean calculation to avoid downward bias from background noise.

### Data visualization

In addition to the specific methods described for PhIP-Seq and protein-level analyses, the following packages were used for analysis and visualization: tidyverse (50), ggpubr (51), and ggrepel (52).

### ELISA

#### Recombinant antigen ELISA.

Selected whole-protein antigens identified from the PhIP-Seq analysis were orthogonally validated using ELISA. Recombinant antigens were either obtained commercially — human COL1A1 (NM_000088, Origene) and PPP1R12B (NM_032104, Origene) — or produced recombinantly using Expi293 cells as the expression host and purified using standard nickel affinity and size exclusion chromatography (CD226; NM_006566, Callaghan Innovation). For the whole-protein ELISAs, Nunc immunoplates (Sigma-Aldrich) were coated with recombinant antigen at either 2 μg/mL (COL1A1, PPP1R12B), or 5 μg/mL (CD226) in phosphate-buffered saline (pH 7.4) (PBS) and left overnight at 4°C. Following washing with PBS/0.1% Tween 20, wells were blocked with either PBS/0.5% human serum albumin (HSA) (Sigma-Aldrich) (COL1A1, PPP1R12B) or PBS/1% bovine serum albumin (BSA) (MP Biomedicals) (CD226) for 1 hour at 37°C. After a further washing, serum was added at a 1:200 dilution in either PBS/0.1% HSA (COL1A1, PPP1R12B) or PBS/1% BSA (CD226) for 1 hour at 37°C. IgG binding was detected using goat anti–human IgG conjugated with horseradish peroxidase (ab97225, Abcam) and binding was detected with 3,3′,5,5′-tetramethylbenzidine and stopped with 1 M hydrochloric acid. The optical density (450 nm) was measured using an EnSight absorbance reader. Two internal controls (with high and low ELISA absorbance values) per antigen were included on each plate with the acceptance criteria being an interassay coefficient of variation (CV) of below 15%. ELISAs were performed using the same samples used in PhIP-Seq (Table 1).

#### Peptide ELISA.

For further validation of PPP1R12B, 2 peptides (45 amino acids in length) aligning with the detected 90–amino acid fragment identified by PhIP-Seq were synthesized (Genscript Biotech). The peptides were coated at 5 μg/mL in carbonate/bicarbonate buffer (pH 9.6) on Nunc immunoplates (Sigma-Aldrich) and incubated at 37°C for 3 hours before being left overnight at 4°C. Following 3 washes with PBS/0.1% Tween 20, serum diluted 1:100 in PBS/0.1% Tween 20 was added and incubated at 37°C for 2 hours. Detection of IgG binding was carried as per the recombinant antigen ELISA above, and the same acceptance criterion was applied (interassay CV < 15% for internal plate controls). The operating threshold cutoff for the ELISA was determined in the discovery cohort (absorbance, optical density [OD] scale) using pROC’s “closest.topleft” criterion, i.e., the cutoff point that minimizes (1 – sensitivities)^2^ + (1 – specificities)^2^ (53). This yielded an OD cutoff of 0.737, which applied unchanged to the validation cohort. Performance in the validation cohort (ARF vs. combined healthy and Strep A pharyngitis controls) was evaluated at this prespecified OD cutoff. Sensitivity, specificity, and accuracy were accompanied by 95% CIs from a 2,000-iteration class-stratified bootstrap (quantile method). ROC AUC and its 95% CI were estimated by the DeLong method.

### Statistics

Statistics for each analyses are provided in detail in the relevant sections above. In summary, for per-donor PhIP-Seq peptide calling each peptide 1-sided (right-tailed) *P* values were derived per donor and adjusted within donor by BH and a BH-adjusted *P* value of 0.10 or less considered significant. For case-control differential peptide analysis, count-level modeling used DESeq2 with BH correction across peptides (significance at FDR < 0.05). Finally, for pathway enrichment, GO enrichment used Metascape’s default multiple-testing correction; enriched terms are reported at FDR *q* less than 0.05.

### Study approval

Human studies were reviewed and approved by the Health and Disability Ethics Committee (HDEC), Ministry of Health, Wellington, New Zealand (RF RISK: 14/NTA/53; Strep A skin and throat infection study: 17/NTA/262). All participants (or their proxies) provided written informed consent.

### Data availability

The original contributions presented in the study are included in the article/supplemental material. Values for all data points in graphs are reported in the Supporting Data Values file. Further inquiries can be directed to the corresponding authors.

## Author contributions

RM, NW, MGB, UL, and NJM designed and initiated the study. RM, LHC, TJOD, EM, CS, and UL designed and performed the experiments and RM, UL, TJOD, LHC, and RM analyzed the results. WJM and FCM provided cultural governance. NJM and RM obtained funding. RM, LHC, and NJM interpreted the results. RM and NJM co-wrote the original manuscript, and all authors read and reviewed the manuscript.

## Funding support

- Cure Kids New Zealand (project 3720979).
- Maurice Wilkins Centre for Molecular Biodiscovery (project 3716490).
- University of Auckland Doctoral Scholarship (to LC).
- Health Research Council (HRC) Rheumatic Fever Research Partnership grant (for the RF RISK study and the Strep A skin and throat study).

## Supplementary Material

Supplemental data

Supplemental data set 1

Supporting data values

## Figures and Tables

**Table 1:**
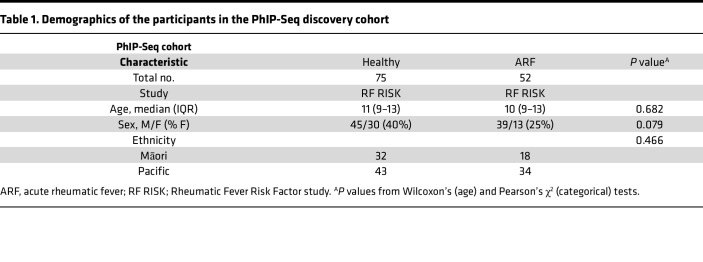
Demographics of the participants in the PhIP-Seq discovery cohort

## Version 1 11/18/2025

In-Press Preview

## Version 2 01/09/2026

Electronic publication
