# Supplementary material for: PhIP-Seq uncovers marked heterogeneity in acute rheumatic fever autoantibodies
Source: JCI Insight. 2025 Nov 18;11(1):e196619. doi: 10.1172/jci.insight.196619 (PMC12890473; doi:10.1172/jci.insight.196619)
Supplement: Supplemental data [file jciinsight-11-196619-s074.pdf]

**Validation cohort**			
**Characteristic**	Healthy	ARF	Strep A+ pharyngitis
Total No.	16	36	54
Study	RF RISK	RF RISK	GAS skin/throat
Age, Median (IQR)	12.5 (11-13)	11.5 (10-13)	9 (7-11)
Sex, M/F (% F)	11/5 (31%)	21/15 (42%)	32/22 (41%)
Ethnicity			
Māori	5	13	18
Pacific	11	21	20
Other[A]	0	2	16

Supplementary Table 1| Demographics of the participants included in the validation cohort|

Abbreviations: **ARF**, Acute Rheumatic Fever; **RF RISK**; Rheumatic Fever Risk Factor study; **Strep A+ pharyngitis**, group A Streptococcus-culture positive pharyngitis.
[A] Participants belonging to ethnicity group “Other” (non-Māori and non-Pacific ethnic background)

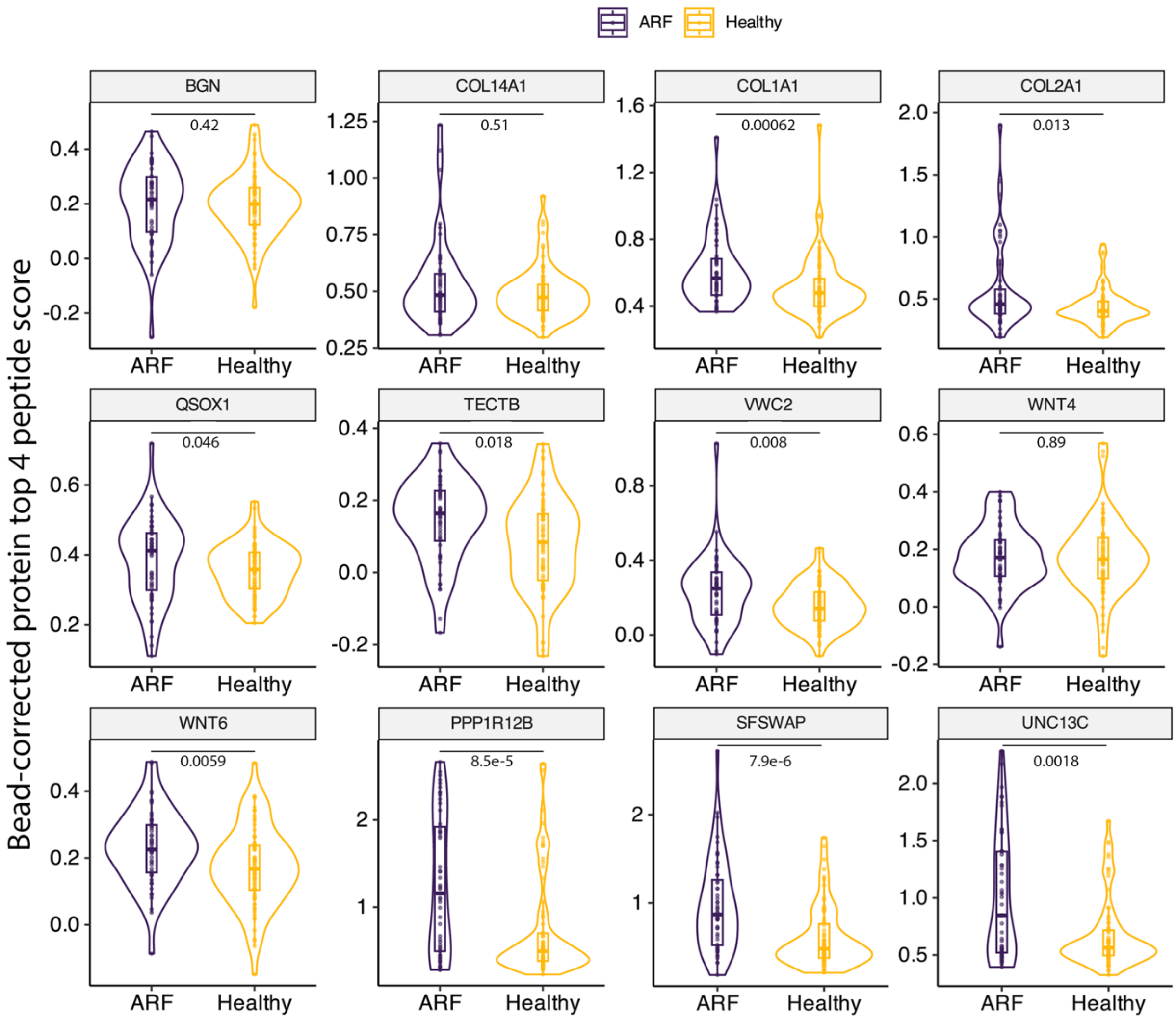

Supplementary Figure 1|Autoantibodies to collagen pathway proteins and PPP1R12B| Violin plots showing the distribution of autoantibody reactivity for collagen-associated proteins as well as the top 3 proteins identified by sPLS-DA (PPP1R12B, SFSWAP and UNC13C proteins) in ARF cases (purple) and healthy controls (gold), using sum of top 4 proteins as described in methods. Boxplots indicate the median and interquartile range, with individual samples overlaid as points. Data are shown on independent y-axis scales for each protein. Statistical significance was determined for each protein using the Wilcoxon rank-sum test. P-values indicated in black text.

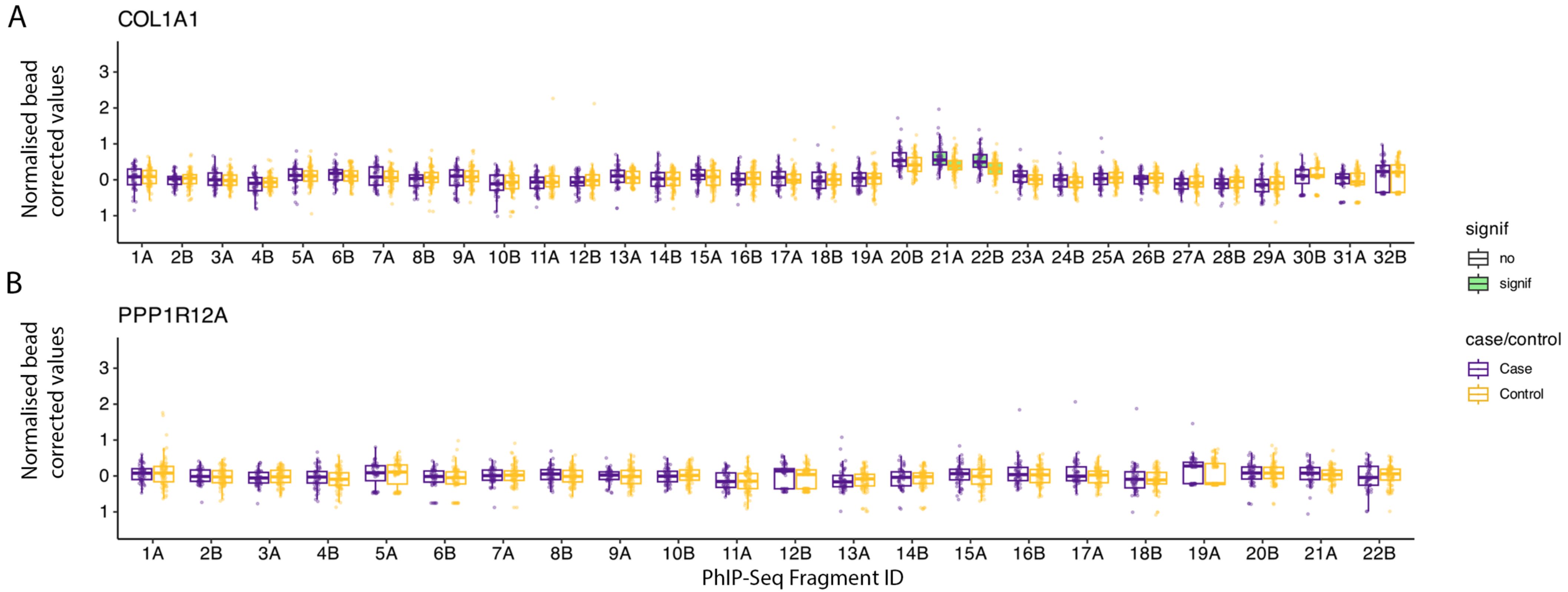

Supplementary Figure 2|Full protein coverage PhIP-Seq data| Individual peptide enrichment for **(A)** COL1A1 and **(B)** PPP1R12A peptides in ARF (purple) vs controls (gold); significantly enriched peptides (only present in COL1A1) from DEseq2 analysis highlighted green. Boxplots indicate the median and interquartile range, with individual samples overlaid as points.
